# Development and assessment of PharmaCheck: an electronic screening tool for the prevention of twenty major adverse drug events

**DOI:** 10.1186/s12911-022-01885-8

**Published:** 2022-05-31

**Authors:** Christian Skalafouris, Jean-Luc Reny, Jérôme Stirnemann, Olivier Grosgurin, François Eggimann, Damien Grauser, Daniel Teixeira, Megane Jermini, Christel Bruggmann, Pascal Bonnabry, Bertrand Guignard

**Affiliations:** 1 Pharmacy, Geneva University Hospitals, Rue Gabrielle-Perret-Gentil 4, 1205 Geneva, Switzerland; 2 General Internal Medicine Division, Geneva University Hospitals, Rue Gabrielle-Perret-Gentil 4, 1205 Geneva, Switzerland; 3 Information Systems Department, Geneva University Hospitals, Rue Gabrielle-Perret-Gentil 4, 1205 Geneva, Switzerland; 4 Institute of Pharmaceutical Sciences of Western Switzerland (ISPSO), School of Pharmaceutical Sciences, University of Geneva, Geneva, Switzerland

**Keywords:** Clinical pharmacy, Clinical decision support system (CDSS), Rule-based system, Clinical rules

## Abstract

**Background:**

Adverse drug events (ADEs) can be prevented by deploying clinical decision support systems (CDSS) that directly assist physicians, via computerized order entry systems, and clinical pharmacists performing medication reviews as part of medical rounds. However, physicians using CDSS are known to be exposed to the alert-fatigue phenomenon. Our study aimed to assess the performance of PharmaCheck—a CDSS to help clinical pharmacists detect high-risk situations with the potential to lead to ADEs—and its impact on clinical pharmacists’ activities.

**Methods:**

Twenty clinical rules, divided into four risk classes, were set for the daily screening of high-risk situations in the electronic health records of patients admitted to our General Internal Medicine Department. Alerts to clinical pharmacists encouraged them to telephone prescribers and suggest any necessary treatment adjustments. PharmaCheck’s performance was assessed using the intervention’s positive predictive value (PPV), which characterizes the proportion of interventions for each alert triggered. PharmaCheck’s impact was assessed by considering clinical pharmacists as a filter for ruling out futile alerts and by comparing the final clinical PPV with a pharmacist (the proportion of interventions that led to a change in the medical regimen) to the final clinical PPV without a pharmacist.

**Results:**

Over 132 days, 447 alerts were triggered for 383 patients, leading to 90 interventions (overall intervention PPV = 20.1%). By risk class, intervention PPVs made up 26.9% (n = 65/242) of abnormal laboratory value alerts, 3.1% (4/127) of alerts for contraindicated medications or medications to be used with caution, 28.2% (20/71) of drug–drug interaction alerts, and 14.3% (1/7) of inadequate mode of administration alerts. Clinical PPVs reached 71.0% (64/90) when pharmacists filtered alerts and 14% (64/242) if they were not doing it.

**Conclusion:**

PharmaCheck enabled clinical pharmacists to improve their traditional processes and broaden their coverage by focusing on 20 high-risk situations. Alert management by pharmacists seemed to be a more effective way of preventing risky situations and alert-fatigue than a model addressing alerts to physicians exclusively. Some fine-tuning could enhance PharmaCheck's performance by considering the information quality of triggers, the variability of clinical settings, and the fact that some prescription processes are already highly secured.

## Background

Medications themselves are one of the most frequent sources of adverse events causing injuries to patients. These events are characterized as adverse drug events (ADEs), and they originate from adverse drug reactions (due to side effects or allergic reactions) or medication errors (MEs) [1]. ADEs may account for about 19% of all the injuries to hospitalized patients, in addition to their economic burden [2–4]. Contrary to non-preventable ADEs (i.e., adverse drug reactions), MEs leading to ADEs are ‘preventable’ when measures can reduce their incidence [1]. MEs can occur at any stage in the treatment process, from prescription to administration, and lead to ADEs [5]. Thus, about half of ADEs could be prevented, particularly using strategies to improve prescription safety [6–8].

Combining a computerized physician order entry (CPOE) system with a clinical decision support system (CDSS) is an effective way of ensuring drug prescription safety [9]. CDSS link information from patients’ electronic health records (EHRs) to knowledge databases and deliver information to improve the quality of medication prescriptions [10]. When coupled with CPOE, a CDSS can produce reminders or alerts to prevent any act of commission or omission that might lead to ADEs. Although this approach has shown some effectiveness in leading to changes in drug treatments, it does have some limitations. Alert override rates vary from 60 to 90% despite significant relevance [11–13]. Several factors may explain a poor adherence rate, especially the cognitive overload that leads to the alert-fatigue phenomenon [14]. Thus, using CDSS helps reduce the incidence of ADEs but is an insufficient way given these limitations.

Clinical pharmacy services contribute to reducing MEs and ADEs through a variety of interventions (e.g., medication reconciliation at admission/discharge, medication review and participation in medical rounds) [15, 16]. In many European countries, clinical pharmacy services are still somewhat scarce: a 2010 survey indicated that only about 40% of hospital pharmacies offered clinical pharmacy services to their colleagues, with great disparities between countries (from 3.6% to 79.2%) [17]. Such disparities also exist in Switzerland. Only 15.0% of hospital pharmacists were assigned to clinical pharmacy duties, with 9.9% to 27.4% full-time equivalent pharmacists allocated to clinical pharmacy activities. These values reflect a lack of human resources available to provide quality pharmaceutical care to an aging, polymedicated population with chronic diseases [17, 18]. In our hospital, pharmaceutical resources are insufficient to attend to every patient requiring a medication review. A previous study indicated that one clinical pharmacist could perform medication reviews for 15 patients per day [19]. Our present resources would only allow us to cover a small portion of all inpatients.

As mentioned above, computerization is supposed to help meet the two major challenges facing hospitals: providing better care and lowering costs. Furthermore, we hypothesized that the performance of CDSS in preventing ADEs could be improved if they were managed by clinical pharmacists. It might also increase pharmacists’ scope for action by making it possible for them to identify greater numbers of high-risk situations that might lead to an ADE. Our study aimed to assess the performance of a CDSS dedicated to clinical pharmacists for the detection of high-risk situations potentially leading to an ADE as well as the tool’s impact on the activity of clinical pharmacists.

## Methods

### Setting

In the 2,000-bed Geneva University Hospitals, integrated computerized patient records provide administrative and demographic data, structured hospitalization reports (e.g., forms, discharge letters, progress notes, radiology reports, nursing reports), laboratory values, and CPOE. A CDSS supports CPEO with suggestions regarding in-label and default values for drugs, dosages, and routes of administration. The CDSS also performs several checking procedures (e.g., for the presence of a drug allergy, medication duplication, drug–drug interactions, and overdosage). Alerts are displayed during the prescription process and must be acknowledged by the physician to ensure that the medical order is validated. Clinical pharmacy activities are carried out by the three clinical pharmacists in the General Internal Medicine Department. They make a total of three to five visits to the wards per week, covering an average of 45 patients (about 20% of admitted patients). There was no CDSS dedicated to clinical pharmacists before this study.

### PharmaCheck’s development and clinical rules selection

After analyzing the market for CDSS dedicated to clinical pharmacists and meetings with service providers, we decided to develop an in-house solution integrated with our institutional EHRs. Thus was born PharmaCheck, with two major advantages. Firstly, the flexibility offered by in-house development allowed us to follow our specifications more closely and interface better with our EHR’s different modules (e.g., CPOE, laboratory services). Secondly, developing an in-house solution cost less than the commercial solutions offered via business models requiring annual licensing. PharmaCheck emerged from a collaboration between our hospital’s pharmacy and the Information Systems Department, and also resulted in the research pharmacist acquiring programing skills for the creation of electronic queries.

**Fig. 1:**
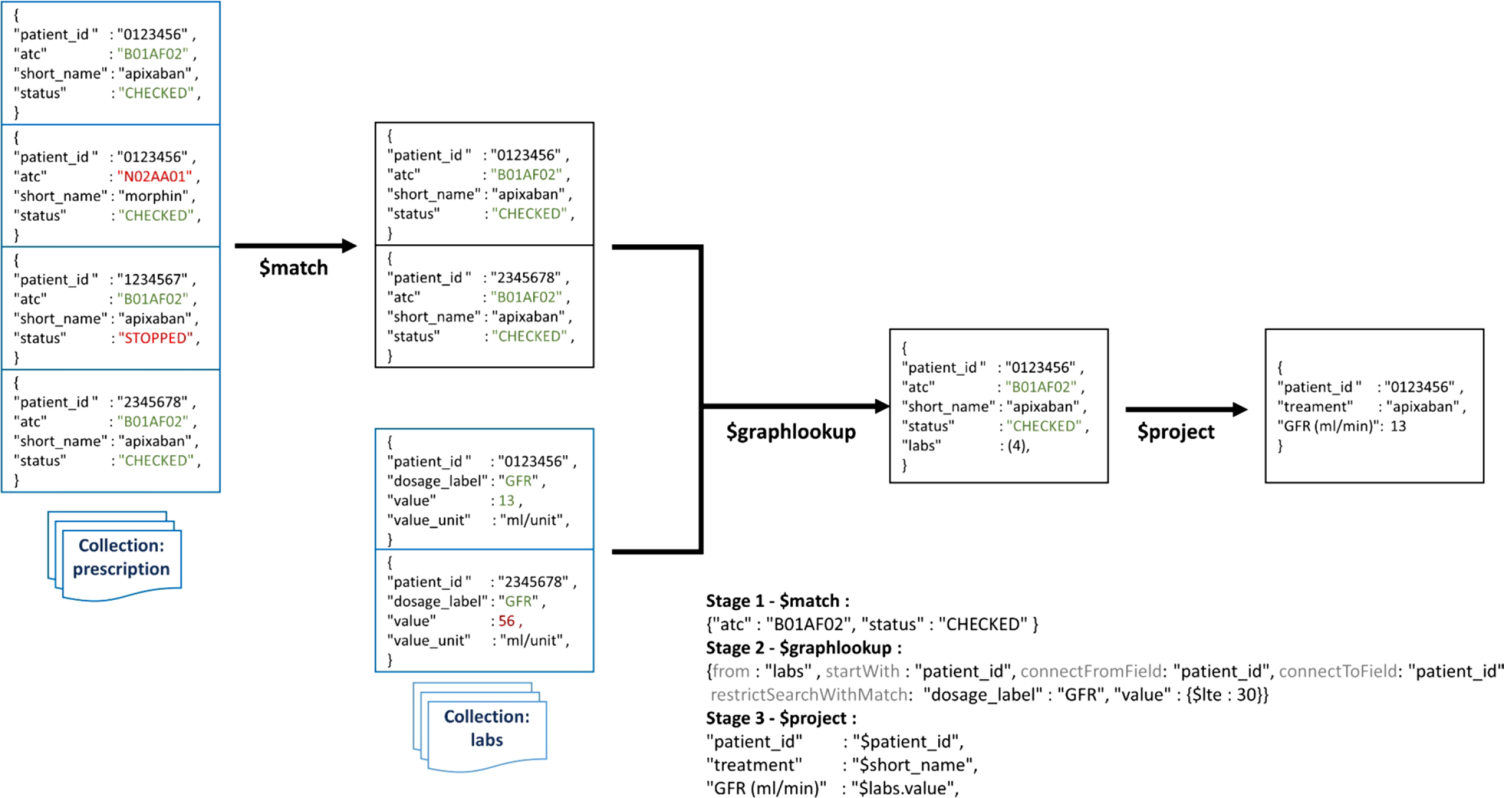
Example of an aggregation pipeline. This three-stages aggregation query describes how EHR are filtered to identify patients with at least one apixaban prescription and an estimated glomerular renal function (GFR) lower than 30 mL/min: First Stage: The $match stage filters the prescription database to identify the medication by its anatomical therapeutic chemical (atc) code and its status. Only prescriptions that concern apixaban (atc equal to "B01AF02″) and that have be signed and are still active (status equal to”CHECKED”) pass on to the next stage; Second Stage: The $graphlookup stage lookups laboratory values with a publication date of less than 30 days (step not shown in the example) for the patients identified with the first stage. Thus, lookup stage is performed in the list of all recent published laboratory data concerning patient(s) whose identifier ("patient_id") has been isolated in the first step and restricted to analysis with a dosage_label equal to “GFR” and a value lower than or equal to 30 (mL/min); Third stage: The $project stage enables the construction of the table of aggregated results with patient identifier (patient_id), medical order description (treatment), and eGFR value (GFR (ml/min))

In our institution, patients’ EHRs are stored in a document-based data warehouse (DPI-DATA) that is consultable using MongoDB aggregation operations. Aggregation operations group values from multiple sources together and can perform a variety of operations on those grouped data to return a single result [20]. An ‘aggregation pipeline’ approach was used so that documents enter a multi-stage pipeline that transforms them into an aggregated result. Aggregation operations were built and tested using Studio 3 T software for MongoDB (version 2019.5.1) and involved structured data (drug prescriptions, laboratory values, vital signs, demographics) and unstructured data (medical problems) [21]. PharmaCheck was constructed as a clinical, rules-based system. Accordingly, knowledge is specified via facts and IF–THEN rules, and *modus ponens* was used as the underlying inference method for deriving new conclusions from existing knowledge [22]. An illustration of an aggregation pipeline is shown in Fig. 1.

**Fig. 2:**
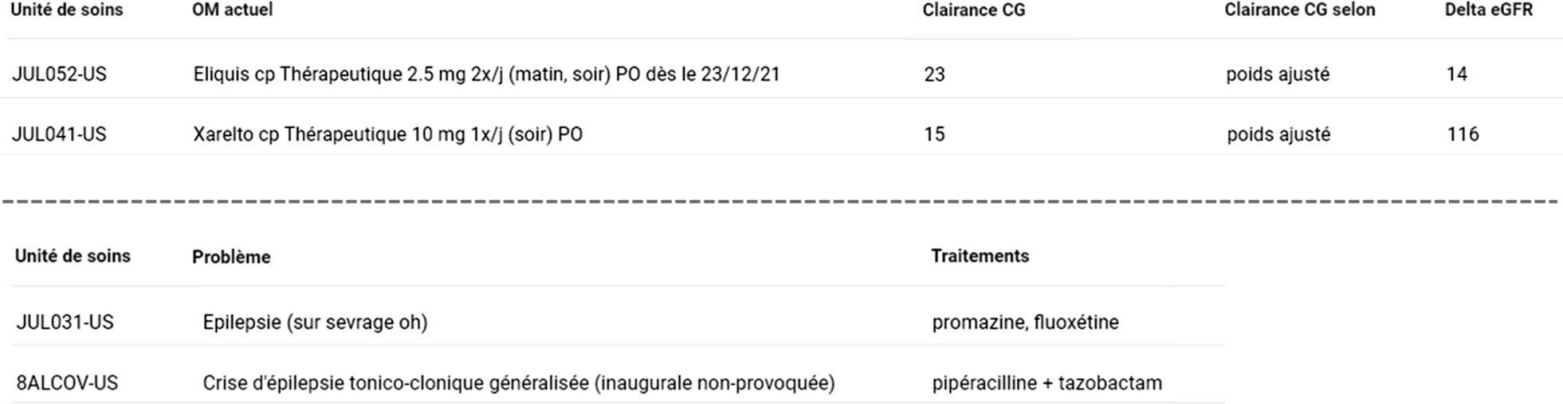
PharmaCheck screenshots. Top screenshot: This example presents the results produced by a PharmaCheck alert when prescribing certain anticoagulants (apixaban—Eliquis®, rivaroxaban—Xarelto®) in the presence of a creatinine clearance below 30 mL/min. The first columns show patient name, age, episode-of-care number (hidden in screenshot) The following columns show inpatient unit (“Unité de soins”), and current medical order (“OM actuel”) related to the anticoagulant. Then the creatinine clearance is calculated using the Cockcroft and Gault formula (“Clairance CG”), taking into account the patient’s adjusted weight (“Clairance CG selon”). PharmaCheck enables patient’s adjusted weight calculation using mathematical operations that consider sex, creatinine values, weight, and height. Similarly, further mathematical operations are performed on available estimated renal function values (“Delta eGFR”). These operations calculate the progression of renal function (14% and 116% increases, respectively) between the last two eGFR measurements. Bottom screenshot: This example presents the results produced by a PharmaCheck alert when prescribing drugs lowering seizure threshold (“Traitements”) in patients with a history of epilepsy characterized by the French world "épilepsie" in patient notes (“Problème”)

For each high-risk situation assessed, PharmaCheck renders a table presenting the patient’s characteristics and all the trigger values. According to the situation assessed, other informative values that might ease clinical decision-making are displayed (e.g., previous laboratory values, medication history). The CDSS interface is presented in Fig. 2. The triggering elements and informative values associated with each clinical rule are presented in Table 1.

**Table 1:** Description of the twenty clinical rules

Description of the clinical rule	Triggers	Other informative values displayed
*Drug prescription with an abnormal lab value*		
Prescription of a DOAC in the presence of an acute renal failure^1^	Active prescription: apixaban or dabigatran or edoxaban or rivaroxaban Laboratory value: ClCG ≤ 30 mL/min (computed with lowest body weight (between measured and ideal body weight))	Prescribed strong inducers/inhibitors of P-gp/CYP3A4; Previous creatinine values
Prescription of colchicine in the presence of an acute renal failure^1^	Active prescription: colchicine Laboratory value: ClCG ≤ 30 mL/min (computed with lowest body weight (between measured and ideal body weight))	Prescribed strong inducers/inhibitors of P-gp/CYP3A4; History of creatinine values
Prescription of morphine in the presence of an acute renal failure^1^	Active prescription: morphine Laboratory value: ClCG ≤ 15 mL/min (computed with lowest body weight (between measured and ideal body weight))	Previous creatinine values
Prescription of metformin in the presence of an acute renal failure^1^	Active prescription: metformin Laboratory value: ClCG ≤ 30 mL/min (computed with lowest body weight (between measured and ideal body weight))	Previous creatinine values
Prescription of metformin in the presence of hyperlactatemia^2^	Active prescription: metformin Laboratory value: lactatemia ≥ 5.0 mmol/L	Previous lactate values
Prescription of heparin in the presence of a thrombopenia^2^	Active prescription: LMWH, UFH Laboratory value: platelets ≤ 50 G/L	Previous LMWH and/or UFH prescriptions Previous platelet count
Prescription of digoxin in the presence of dyskaliemia^2^	Active prescription: digoxine Laboratory value: kaliemia ≤ 3.5 mM or kaliemia ≥ 5.5 mM	Previous digoxin prescriptions Previous potassium values
Prescription of digoxin in the presence of high rate of digoxinemia^2^	Active prescription: digoxine Laboratory value: digoxinemia ≥ 3 nM	Previous digoxin prescriptions Previous digoxin values
Prescription of a blood-glucose-lowering drug in the presence of hypoglycemia^2^	Active prescription: blood-glucose-lowering drug (ATC A10B) + insulins (exclusion of PRN prescriptions) Laboratory value: glycemia ≤ 4 mM	Previous glucose values
Prescription of VKA in the presence of a supra-therapeutic INR^3^	Active prescription: acenocoumarol, fluindione, warfarin, phenprocoumon Laboratory value: INR ≥ 4	Previous doses of VKA; Previous INR values
Prescription of vancomycin in the presence of high rate of vancomycinemia^3^	Active prescription: vancomycin Laboratory value: vancomycinemia ≥ 25 mg/L	Previous vancomycin levels
Prescription of gentamicin with supratherapeutic gentamycine rate Prescription of tobramycin with supratherapeutic tobramycin rate Prescription of amikacin with supratherapeutic amikacin rate^3^	Active prescription: gentamicin or tobramycin or amikacin Laboratory value: gentamicinemia ≥ 1 mg/L; tobramycinemia ≥ 1 mg/L; amikacinemia ≥ 5 mg/L	Previous gentamicin, tobramycin, amikacin levels
*Medication contraindicated or to be used with caution*		
Anticholinergic drugs and some comorbidities (e.g., dementia, urinary retention, constipation) [53, 54]	Active prescription: acepromazine and/or aminoalkyl ethers and/or and/or antazoline and/or anticholinergic agents and/or azatadine and/or bamipine and/or buclizine and/or buclizine, combinations and/or carbamazepine and/or chlorcyclizine and/or chlorpromazine and/or cinnarizine + combinations and/or cinnarizine and/or clozapine and/or clozapine and/or cyamemazine and/or cyclizine and/or cyclizine, combinations and/or cyproheptadine and/or deptropine and/or dimetindene and/or disopyramide and/or drugs for functional gastrointestinal disorders and/or drugs for urinary frequency and incontinence and/or fluphenazine and/or hydroxyzine + combinations and/or hydroxyzine and/or hyoscyamine and/or levomepromazine and/or loxapine and/or mebhydrolin and/or meclozine and/or meclozine, combinations and/or nefopam and/or non-selective monoamine reuptake inhibitors and/or oxatomide and/or oxcarbazepine and/or paroxetine and/or pethidine and/or phenindamine and/or phenothiazine derivatives and/or pimethixene and/or pimozide and/or piritramide and/or pizotifen and/or propantheline and/or pyrrobutamine and/or pyrrobutamine, combinations and/or quetiapine and/or scopolamine (hyoscine) and/or scopolamine and/or substituted alkylamines and/or substituted ethylene diamines and/or synthetic anticholinergic agents in combination with analgesics and/or synthetic anticholinergics, esters with tertiary amino group and/or synthetic anticholinergics, quaternary ammonium compounds and/or synthetic antispasmodics, amides with tertiary amines and/or thenalidine and/or thenalidine, combinations and/or thioridazine and/or thiothixene and/or tizanidine Active problem (indicated in the admission note): dementia, acute confusional state, confusion, urinary globe, prostatism, urine retention, acute angle-closure glaucoma, cardiac conduction disorder	Medication history (extracted from admission note)
Drugs potentially lowering seizure threshold and epilepsy or history of seizure [54]	Active prescription: anticholinergic agents and/or antihistamines for systemic use and/or azithromycin and/or beta-lactam antibacterials, penicillins and/or bupropion and/or busulfan and/or calcineurin inhibitors and/or carmustine and/or chlorambucil and/or chlorpromazine and/or clonidine and/or clozapine and/or cyamemazine and/or disopyramide and/or domperidone and/or drugs for urinary frequency and incontinence and/or enflurane and/or ephedrine and/or ephedrine and/or flumazenil and/or foscarnet and/or ganciclovir and/or h2-receptor antagonists and/or haloperidol and/or hydroxyzine and/or ketamine and/or ketoconazole and/or levomepromazine and/or lidocaine and/or lithium and/or loxapine and/or mefloquine and/or methotrexate and/or methylphenidate and/or metronidazole and/or midecamycin and/or muscle relaxants, centrally acting agents and/or nefopam and/or non-selective monoamine reuptake inhibitors and/or other beta-lactam antibacterials and/or oxetorone and/or oxybutynin and/or pentazocine and/or pethidine and/or phenothiazines with aliphatic side-chain and/or phenothiazines with piperazine structure and/or phenylpropanolamine and/or pimozide and/or piritramide and/or pizotifen and/or propantheline and/or pyrimethamine and/or quinolone antibacterials and/or scopolamine and/or selective serotonin reuptake inhibitors and/or synthetic anticholinergic agents in combination with analgesics and/or terbutaline and/or theophylline and/or tramadol and/or vincristine and/or Active problem (indicated in the admission note): epilepsy	Medication history (extracted from admission note)
NSAID and some comorbidities (e.g., renal failure, heart failure) [54]	Active prescription: NSAID (ATC = M01A) and/or metamizole and metamizole, combinations excl. psycholeptics and/or metamizole, combinations with psycholeptics Active problem (indicated in the admission note): chronic renal failure and/or heart failure and/or myocardial infarction and/or gastric ulcer and/or duodenal ulcer and/or gastroduodenal ulcer	Medication history (extracted from admission note)
*Drug-drug interactions*		
Co-prescription of 2 anticoagulants	Active prescription: acenocoumarol and/or apixaban and/or dabigatran and/or fluindione and/or LMWH and/or prophencoumon and/or rivaroxaban and/or UFH and/or warfarin (VKA and LMWH or UFH excluded)	
Co-prescription of 2 serotoninergic drugs [55]	At least two of the following active prescriptions: amitriptyline and/or bromocriptine and/or bupropion and/or buspirone and/or cabergoline and/or carbamazepine and/or citalopram and/or clomipramine and/or clozapine and/or dextromethorphane and/or dihydroergotamine and/or dosulepine and/or doxepine and/or eletriptan and/or ergotamine and/or escitalopram and/or fentanyl and/or fluoxetine and/or fluvoxamine and/or haloperidol and/or imipramine and/or isoniazide and/or lamotrigine and/or linezolide and/or lithium and/or maprotiline and/or methadone and/or metoclopramide and/or mianserine and/or mirtazapine and/or moclobemide and/or naratriptan and/or nortriptyline and/or olanzapine and/or ondansetron and/or oxycodone and/or paroxetine and/or pergolide and/or pethidine and/or quetiapine and/or risperidone and/or rizatriptan and/or selegiline and/or sertraline and/or sibutramine and/or sumatriptan and/or tramadol and/or trazodone and/or trimipramine and/or valproate and/or venlafaxine and/or zolmitriptan	Medication history (extracted from admission note)
*Inadequate administration mode*		
Scheduling methotrexate doses less than 7 days apart	Nursing scheduling of 2 MTX doses in a ≤ 7 days interval	
Prescription of intravenous potassium chloride at too high a flow rate	Active prescription: parenteral intravenous potassium chloride flow rate > 10 mmol/hour	
Prescription of intravenous potassium chloride at too high a concentration	Active prescription: parenteral intermittent intravenous chloride at a concentration > 0.08 mmol/mL; parenteral continuous intravenous chloride at a concentration > 1.00 mmol/mL	

Each of the twenty clinical rules is classified according to four risk categories (drug prescription with an abnormal laboratory value; medication contraindicated or to be used with caution regarding clinical context; drug–drug interaction; inadequate mode of administration). Drug prescriptions with abnormal laboratory values are subdivided into three sub-categories (1: drug prescription in the presence of renal failure; 2: drug prescription combined with a supra-therapeutic serum level; 3: drug prescription combined with an abnormal laboratory value indicating an overdosage or a risk of an adverse effect). Trigger factors are described for each clinical rule (prescribed drug, mode of drug administration, laboratory value, patient problem), as are all the displayed elements extracted from the computerized patient record and intended to facilitate decision-making

*DOAC* direct oral anticoagulant, *VKA* vitamin K antagonist, *NSAID* non-steroidal anti-inflammatory drug, *ClCG* estimated creatinine clearance using the Cockcroft–Gault formula, *LMWH* low molecular weight heparin, *UFH* unfractionated heparin, *ATC* anatomical therapeutic chemical, *PRN* as needed, *INR* international normalized ratio, *MTX* methotrexate, *P-gp* permeability glycoprotein, *CYP3A4* cytochrome P450 3A4

The clinical rules for screening high-risk situations that might lead to major adverse drug events were selected using a two-stage process. This involved a clinical pharmacist (BG), with over ten years of experience in internal medicine, and the post-graduate research pharmacist (CS) responsible for programming PharmaCheck:

Firstly, we assessed (BG) situations described as ‘critical’ in international guidelines—or treated as such in an equivalent screening tool used by the Hôpital du Valais (another Swiss hospital) to deal with extremely critical situations [23–25]. As these queries were intended to be tested in the General Internal Medicine Department, critical situations corresponding to patients’ prescription profiles were selected. This department treats adult patients with various specific pathologies (heart failure, renal failure, decompensated diabetes, etc.) and we focused on fairly general situations that might concern any patient. Secondly, we selected (CS) clinical rules that could be transposed into our CDSS and based on triggers identifiable from electronic queries. Triggers were tested on data from past hospitalizations stored in our clinical data warehouse over the past 20 years. We checked their effectiveness in detecting the constituent elements of each electronic query (e.g., for the rule “prescription of intravenous potassium chloride at too high a flow rate”, tests were conducted to validate this condition for detecting potassium chloride prescriptions and then for detecting an administration flow rate greater than 10 mmol/hour). Finally, 20 clinical rules were created for 20 high-risk situations.

Moreover, in this research project, we were interested in testing technical validity of rules that aggregated data from various sources (labs, vital signs, medical problems, administration modalities, frequency of dispensing by the nurse). Thus, clinical rules were divided into four risk categories (Table 1):

1. Drugs prescribed when the patient shows abnormal laboratory values. This risk category included three sub-categories, namely, drug prescriptions with a renal failure, a supra-therapeutic serum level or, an abnormal lab-value relying to an adverse effect
2. Contraindicated medications or medications to be used with caution depending on the clinical context
3. Prescriptions involving a drug–drug interaction
4. Medication prescribed with an inadequate mode of administration

### Study design

PharmaCheck was developed over nine months and evaluated during a seven-month prospective observational study (February to August 2020) including all the patients admitted to the General Internal Medicine Department.

**Fig. 3:**
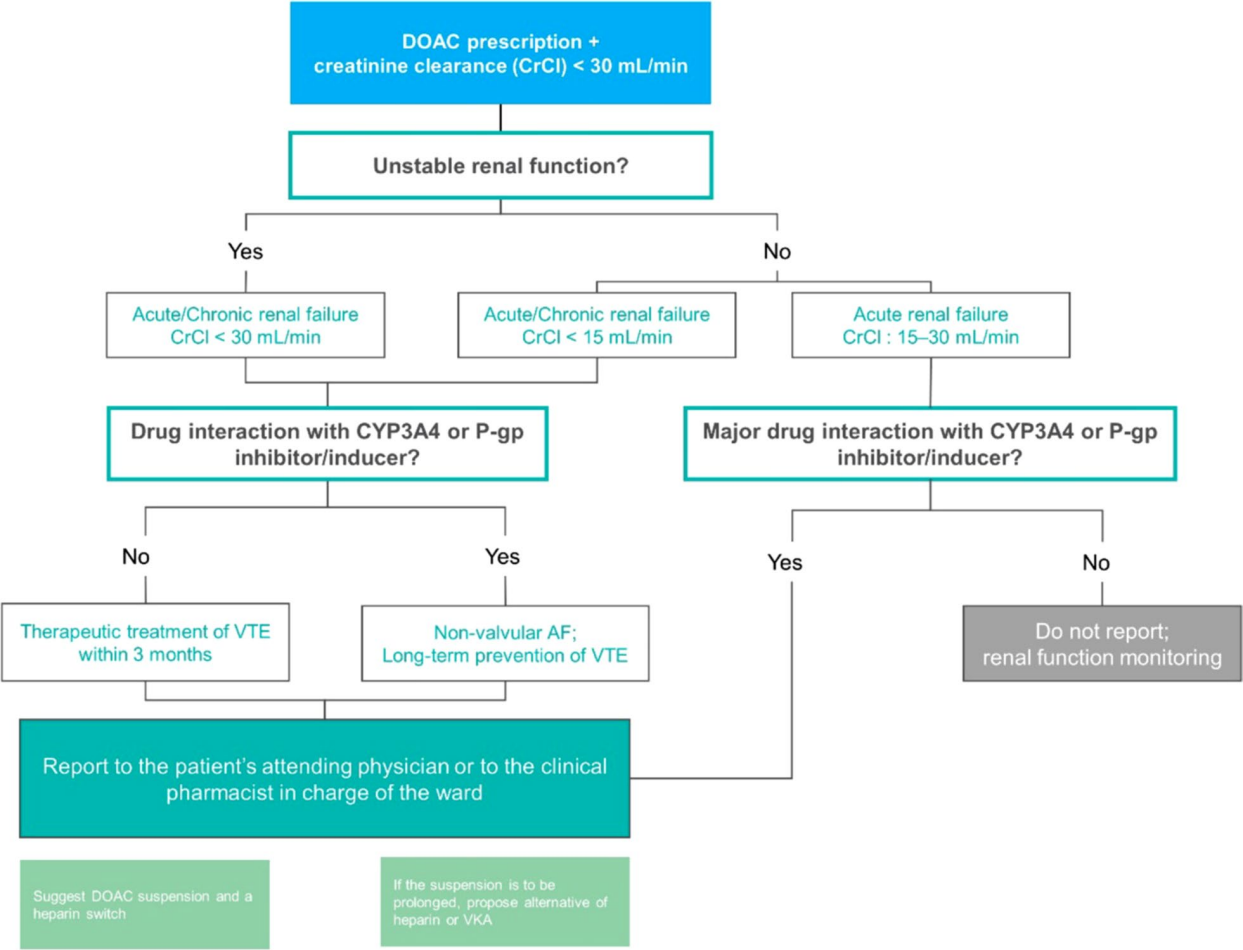
Example of a decisional algorithm. *AF* atrial fibrillation, *CYP3A4* cytochrome P450 3A4, *DOAC* direct anticoagulant oral therapy, *P-gp* permeability glycoprotein, *VTE* venous thromboembolism, *VKA* vitamin K antagonist

Electronic queries were run twice every working day to identify patients exposed to high-risk situations. Each alert was assessed for clinical relevance by a clinical pharmacist to decide whether a suggestion for therapeutic adjustment or additional monitoring should be sent to the prescriber (i.e., via a telephone call or during medical rounds). Decisional algorithms were constructed for the 20 situations to standardize pharmacists’ analyses and intervention each time an alert was triggered (Fig. 3).

A pharmaceutical intervention was considered to have been accepted if it led to a change in patient management within 24 h (e.g., modification of the drug prescription, additional monitoring). We measured the distribution for each alert and a repetitive alert was only counted once if the pharmacist had already dealt with it. An electronic tracking file made it possible to document pharmacists’ assessments of the situation, and decisions to intervene or to follow-up on the situation. The file was shared with the rest of the pharmaceutical team.

PharmaCheck’s ability to detect high-risk situations was assessed by measuring the clinical pharmacy intervention’s positive predictive value (PPV) for each alert:

$$Intervention\;PPV = \frac{number\; of\; pharmaceutical\; interventions}{{number\; of\; alerts\; fired\; off\; with\; PharmaCheck}}$$

The clinical pharmacist’s ability to act as a filter, ruling out futile alerts, was assessed by comparing the final clinical PPV including pharmacists’ interventions with the final clinical PPV without pharmacists. This latter hypothetical value assumed that all the prescription modifications decided upon by the clinical pharmacist would also have been made if the alerts had been sent directly to physicians.

$$Clinical{ }\;PPV\;\left( {with\; pharmacist} \right) = \frac{{number{ }\;of\;{ }prescription\; change}}{{number{ }\;of{ }\;pharmaceutical \;interventions}}$$

$$Clinical \;PPV\;\left( {without\; pharmacist} \right) = \frac{{number{ }\;of\;{ }prescription\; change}}{number\; of\; alerts\; fired\; off\; with\; PharmaCheck}$$

When an intervention was suggested and immediately declined, the reasons why our pharmaceutical suggestion was not followed were recorded after discussing them with physicians. Other suggestions were made to the physician in charge of the patient, who also had to discuss them with other attending physicians before any final decision. In some cases, this resulted in a delayed refusal of our suggestions, somewhat removed from our intervention. In this case, intervention status and reasons why the intervention was declined were recorded by remotely checking the medical EHR (24 to 72 h after the intervention).

### Statistical analysis

Results were expressed as medians (with their interquartile ranges and minimum–maximum values). Non-normally distributed quantitative data were analyzed using non-parametric tests. Differences in the number of pharmaceutical interventions between groups were assessed using the non-parametric Mann–Whitney U test, comparative analyses of the proportions (sex-ratio) were performed using a chi-squared test. Univariate logistic regressions were calculated to identify variables with a strong effect on the probability of a pharmaceutical intervention and its acceptance (binary outcomes). The variables tested were age, number of treatments, and hospital length of stay. A *P*-value less than 0.05 was considered statistically significant. Data analysis was conducted using R software (version 3.6.3).

## Results

PharmaCheck was used to screen 5,466 patients’ EHRs over 132 days. A total of 447 alerts were triggered for 383 patients (7.0% of the patients admitted during the study period). Each day, an average of 3.4 ± 2.0 new alerts were triggered. Patient characteristics and their distribution according to the presence of a pharmaceutical intervention are presented in Table 2; the distribution of alerts by risk category is presented in Table 3.

**Table 2:** Patient characteristics and their distribution according to the presence of a pharmaceutical intervention

Patients characteristics	Total	No intervention	Intervention	*p*-value	*Odd ratio [95CI]*
Number of patients	383	301	82	–	–
Sex ratio = $\frac{{\varvec{n}}{\varvec{u}}{\varvec{m}}{\varvec{b}}{\varvec{e}}{\varvec{r}}\boldsymbol{ }{\varvec{o}}{\varvec{f}}\boldsymbol{ }{\varvec{m}}{\varvec{a}}{\varvec{l}}{\varvec{e}}}{{\varvec{n}}{\varvec{u}}{\varvec{m}}{\varvec{b}}{\varvec{e}}{\varvec{r}}\boldsymbol{ }{\varvec{o}}{\varvec{f}}\boldsymbol{ }{\varvec{f}}{\varvec{e}}{\varvec{m}}{\varvec{a}}{\varvec{l}}{\varvec{e}}}$	1.14	1.23	0.86	0.15	–
Age (years old)	***74*** *IQR: 61–82* *min–max: 17–97*	***73*** *IQR:58–82* *min–max:17–96*	***77*** *IQR: 72–84* *min-max: 19–97*	< 0.001	1.03 [1.01;1.05]
Number of drugs per prescription when alert was fired off	***13*** *IQR: 10–17* *min–max: 1–40*	***13*** *IQR:10–17* *min–max:1–40*	***13*** *IQR:9–18* *min–max:3–30*	0.9	1.0 [0.96;1.03]
Length of stay (days)	**10.5** IQR: 4*–13.5* *min–max: 0–84*	***10*** *IQR:4–13* *min–max:0–84*	***13*** *IQR:6–14.5* *min–max:3–26*	0.57	1.0 [0.96;1.02]

Values in bold represent the medians

*IQR* interquartile range, *95CI* 95% confidence interval

**Table 3:** Distribution of alerts between risk categories

Risk category^1^ Risk sub-category^2^ High-risk situations^3^	Number of alerts	Intervention PPV $\frac{number \;of\; interventions}{{number\; of\; alerts}}$	Clinical PPV with a pharmacist $\frac{number\; of \;accepted\; therapeutic\; suggestions }{{number \;of \;interventions}}$	Clinical PPV without a pharmacist $\frac{number\; of\; accepted\; therapeutic\; suggestions }{{number \;of \;alerts}}$
Drug prescription with an abnormal lab value^1^	242 (54.1%)	26.9% (n = 65)	66.2% (n = 43)	17.8%
Drug prescription in the presence of renal failure^2^	121 (27.1%)	33.9% (n = 41)	70.7% (n = 29)	24.0%
DOAC and acute renal failure^3^	64	39.1% (n = 25)	64.0% (n = 16)	25.0%
Colchicine and acute renal failure^3^	21	28.6% (n = 6)	66.7% (n = 4)	19.0%
Metformin and acute renal failure^3^	20	45.0% (n = 9)	88.9% (n = 8)	40.0%
Morphine and acute renal failure^3^	16	6.3% (n = 1)	100% (n = 1)	6.3%
Drug prescription combined with a supra-therapeutic serum level^2^	42 (9.4%)	26.2% (n = 11)	36.4% (n = 4)	9.5%
VKA and supra-therapeutic INR^3^	36	19.4% (n = 7)	28.6% (n = 2)	5.5%
Vancomycin and supra-therapeutic vancomycin rate^3^	4	75.0% (n = 3)	33.3% (n = 1)	25.0%
Digoxin and supra-therapeutic digoxin rate^3^	1	100.0% (n = 1)	100% (n = 1)	100.0%
Aminoglycosides and supra-therapeutic aminoglycoside rate^3^	1	0.0% (n = 0)	NA	NA
Drug prescription combined with an abnormal lab value^2^	79 (17.7%)	16.5% (n = 13)	77.0% (n = 10)	12.7%
Blood glucose lowering drug and hypoglycemia^3^	30	0.0% (n = 0)	NA	NA
Heparin and thrombopenia^3^	30	13.3% (n = 4)	50.0% (n = 2)	6.7%
Digoxin and dyskalemia^3^	15	46.7% (n = 7)	85.7% (n = 6)	40.0%
Metformin and acute hyperlactatemia^3^	4	50.0% (n = 2)	100% (n = 2)	50.0%
Medication contraindicated or to be used with caution^1^	127 (28.4%)	3.1% (n = 4)	75.0% (n = 3)	2.4%
Anticholinergic drugs and some comorbidities (e.g., dementia, urinary retention, constipation)^3^	66	1.5% (n = 1)	100% (n = 1)	1.5%
Drugs lowering seizure threshold and epilepsy or history of seizure^3^	51	2.0% (n = 1)	0% (n = 0)	0.0%
NSAID and some comorbidities (e.g., renal failure, heart failure)^3^	10	20.0% (n = 2)	100% (n = 2)	20.0%
Drug–drug interaction^1^	71 (15.9%)	28.2% (n = 20)	85.0% (n = 17)	24.0%
Co-prescription of 2 anticoagulants^3^	38	52.6% (n = 20)	85.0% (n = 17)	48.0%
Co-prescription of 2 serotoninergic drugs^3^	33	0.0% (n = 0)	NA	NA
Inadequate mode of administration^1^	7 (1.6%)	14.3% (n = 1)	0.0% (n = 0)	0.0%
Methotrexate scheduled twice in fewer than 7 days^3^	5	0.0% (n = 0)	NA	NA
Intravenous potassium chloride at a flow rate > 10 mmol/hour^3^	2	50.0% (n = 1)	0.0% (n = 0)	0%
Intravenous potassium chloride at a concentration > 40 mmol/L (peripheral catheter) or >80 mmol/L (central catheter)80 mmol/L (central catheter)^3^	0	NA	NA	NA
Total	447	20.1% (n = 90)	71.0% (n = 63)	14.0%

^1^Risk category; ^2^ Risk sub-category; ^3^ High-risk situation

*DOAC* direct anticoagulant oral therapy, *VKA* vitamin K antagonist, *INR* international normalized ratio, *NSAID* non-steroidal anti-inflammatory drug, *PPV* positive predictive value, *NA* non attributed

Fifteen of our 20 clinical rules led to 90 interventions for therapeutic adjustments or additional monitoring (intervention PPV = 20.1%). Most of the clinical rules (12/20) concerned the ‘drug prescription with abnormal laboratory values’ risk category, accounting for more than half of the alerts (54.1%) and with an intervention PPV of 26.9%.We observed the highest intervention PPV (33.9%) in the ‘drugs prescribed in the presence of renal failure’ sub-category, as these alerts are based on the prescription of a drug (direct oral anticoagulant, metformin, colchicine, and morphine) and an estimated level of renal function. A disparity was observed between PPVs of the clinical rules for the ‘drug prescription combined with an abnormal laboratory value’ sub-category (intervention PPV of 17.7%): in all the prescriptions for blood-glucose-lowering drugs associated with hypoglycemia, the situation was already being managed at the time of screening, and the (intervention PPV = 0.0%). Physicians closely monitored heparin prescriptions in the presence of thrombopenia, as most of the patients concerned were still being monitored for hemopathies. Moreover, the likelihood of heparin‐induced thrombocytopenia was very limited and the PPV was low (13.4%). Finally, the intervention PPV was higher in the presence of hypokaliemia under digoxin (46.7%), or hyperlactatemia on metformin (50.0%). The clinical rules in the sub-category of ‘drug prescription combined with a supra-therapeutic level’ were mainly related to the prescription of vitamin K antagonists and supra-therapeutic INR (36 alerts). These alerts induced few interventions (intervention PPV = 19.40%). The second most frequent alert category was the ‘contraindicated medications or medications to be used with caution’ sub-category (28.4% of alerts). However, this was associated with the lowest intervention PPV (3.1%), which may translate into a low specificity clinical rule (since few alerts were considered relevant enough to trigger an intervention with a physician). The absence of intervention was mainly related to false-positive alerts (e.g., ruling out epilepsy or a history of alcohol withdrawal seizures was sufficient to trigger an alert in the presence of a drug lowering the patient’s seizure threshold).

Two clinical rules involving drug–drug interactions were the third reason for triggering an alert, with an intervention PPV of 28.2% (71 alerts led to 20 interventions). However, given the clinical context, it should be noted that only the clinical rule regarding the interaction of two anticoagulant drugs led to pharmaceutical interventions (intervention PPV = 52.6%). In fact, all of these alerts were accompanied by an intervention except in situations involving the misuse of the CPOE (two anticoagulants prescribed simultaneously when they should be alternated and for which the temporal sequence was clearly indicated in the free-text section of the medication order aimed at the nurse). In contrast, pharmacists never intervened for interactions between two serotoninergic drugs, with an increased risk of serotonin syndrome (intervention PPV = 0.0%). In each case, the doses were low to medium, the patient’s clinical context was incompatible with a serotoninergic syndrome, and the treatments were being taken on a long-term basis and were well tolerated. Alerts associated with an inappropriate mode of administration were the least frequently triggered (n = 7) and had the lowest proportional intervention PPV (14.3%).

Sixty-three interventions were accepted by prescribers, leading to a clinical PPV of 71% when alerts were filtered by a clinical pharmacist, which was 5 times higher than when no pharmacist was filtering alerts (clinical PPV without pharmacist = 14.0%). Considering each alert that led to an intervention, the clinical PPV would have been 1.3 to 67 times lower in a model where alerts would have been exclusively handled by physicians. Only a clinical rule that triggered one clinical alert had the same positive predictive value (digoxin combined with a supra-therapeutic digoxinemia rate, clinical PPV = 100.0%) with and without a pharmacist. Patient age was the only variable to affect the probability of a pharmaceutical intervention. Prescribers decided not to follow 27 suggestions for therapeutic adjustments or additional monitoring, mainly because they assessed there was a positive benefit–risk ratio (85.2%, n = 23 cases), but also for unknown reasons (11.1%, n = 3), and because the patient had already been discharged at the time of the intervention (3.7%, n = 1).

## Discussion

We developed a screening tool to detect several high-risk situations with the potential to lead to a major ADE. Once detected, an alert was sent to clinical pharmacists, not directly to prescribers. The present study had two main findings. Firstly, PharmaCheck was relatively effective at detecting the high-risk situations targeted: 447 potential situations were identified during the study period, of which 90 were considered relevant enough to send a suggested intervention to the treating physician (intervention PPV = 20.1%). Secondly, the final clinical PPV including the pharmacist’s intervention was 71%—five times higher than if the pharmacist had not ruled out the non-clinically relevant alerts (clinical PPV without a pharmacist = 14%). By screening 200 EHR daily, PharmaCheck allowed us to intercept high-risk situations that would not otherwise have been identified, or, at the very least, intercepted them more quickly than our previous standard model of medication review that was limited to 45 patients per week. While this project does not offer itself a scientific significant leap, it stills adds new evidence in terms of CDSS development: programming electronic queries with validated results was relatively accessible (with proper training) resulting in a reliable AI (for the domain where it has been developed), adapted to our context.

### PharmaCheck performance in the detection of high-risk situations

In the present study, decisions to intervene appeared to be moderately positively correlated with patient age (the likelihood of pharmaceutical intervention increased with age). There was clear evidence that older patients were proportionately more polymorbid and polymedicated than younger patients, with an increased risk of ADEs and, therefore, triggering alerts and interventions. Older adults’ intrinsic characteristics may help explain this greater likelihood of intervention as this population is exposed to more adverse events (e.g., reduced renal elimination, greater susceptibility to anticholinergic effects) that readily prompt pharmacists to intervene [26–28]. We assumed that the intervention PPV reflected the specificity of a CDSS and, thus, PharmaCheck’s performance. Few studies have analyzed the impact of advanced CDSS dedicated to clinical pharmacists, and intervention PPVs varied from 8 to 51% [29–32]. Although the reasons for non-intervention were not systematically described, some elements help to explain the disparities in these results: logistical limitations (lack of resources to process every alert); technical limitations (low specificity linked to absent data); and limitations associated with workflows (risk situations that physicians have already considered) [29–32]. We identified three factors that may explain the disparities between the intervention PPVs: the nature and informative quality of the trigger elements; the clinical context and the physician’s awareness of the risk situation; and redundancy in electronic safeguards.

#### Nature and information quality of trigger elements integrated into the CDSS

Alerts concerning drug–laboratory interactions were the most numerous and among those that led to the most pharmaceutical interventions, especially in cases of renal failure. PharmaCheck was set to display several values of renal function: Chronic Kidney Disease Epidemiology Collaboration (CKD-EPI), estimated glomerular filtration rate (eGFR), and Cockcroft–Gault (CG) estimated creatinine clearance, calculated using actual and ideal body weight but selecting the lower one [33, 34]. Thus, most alerts were triggered by CG clearance at ideal body weight, whereas our EHRs only display eGFR using the CKD-EPI formula. Automatically providing and comparing these estimates of renal function added value and prompted pharmaceutical interventions. In contrast, clinical rules involving patient-related problems were linked to poor intervention PPV. Triggers characterizing patient-related problems are not commonly structured in an unequivocal way (e.g., using ICD-10 terminology and/or SNOMED CT) [35]. Alternatively, patient-related problems can be targeted using free-text searches through EHRs, but only with uncertain reliability and a sensitivity varying from 1 to 46% [36]. Here, the search for free-text terms in admission notes was not sufficiently specific. We believe that these issues will be partially solved by the General Internal Medicine Department’s recent deployment of a structured patient-problem list, the use of which has led to a clear decrease in free-text entries on several wards over the last three years [37].

#### Clinical context and physicians’ awareness of risk situations

It has been shown that integrating contextual information is a key factor in improving the PPV for medication alerts [38]. Additionally, two of the most significant contextual factors that should help clinicians’ decision-making within a CDSS are the ‘severity of the effect’ and the ‘patient’s clinical condition’, which remain difficult to assess using clinical rules based on explicit criteria. Thus, contextual factors depend mainly on clinicians’ judgment [39]. Clearly, prescribing two anticoagulants should be avoided, given the ‘severity of effect’ and the immediate risk this represents. Interventions were carried out as soon as these situations were presented themselves (with the exception of CPOE misuse, which did not lead to intervention). Identically, an intervention was done in the presence of hypokalemia under digoxin, or hyperlactatemia under metformin, due to the potential severity of the adverse effects and an unfavorable benefit–risk ratio [40, 41]. In contrast, no interventions were carried out for situations considered insufficiently risky considering the context (e.g., low-dose serotonergic drugs), or which were already being monitored (e.g., resugaring following hypoglycemia), or which were subject to enhanced monitoring (e.g., thrombocytopenia). Thus, a significant effort was made to improve ergonomics, to display useful information directly adjacent to alerts, and to contextualize them using patient data (e.g., medication and dosage, previous laboratory values) [42].

#### Redundant safeguards

Clinical rules regarding an inappropriate mode of administration targeted drugs described in the list of ‘never events’ for which the occurrence of an ADE may lead to a life-threatening situation [23]. Thus, methotrexate and potassium chloride were already the targets of priority safety actions (i.e., restrictions in dosage selection). However, deviations from prescriptions were still theoretically possible; for example, a prescription of two separate single doses of methotrexate at an interval of less than 7 days was possible despite a locked-in administration frequency option (once a week) and a duplicate alert trigger. Here, PharmaCheck was used as a complementary strategy to prevent ‘never events’, even though their probability of occurrence was very low, as shown by the low intervention PPV (the only intervention concerned a severe hypokalemia, for which a potassium chloride infusion rate of > 10 mmol/hour was prescribed without any documentation on cardiac monitoring). The same was true for ‘VKA and supra-therapeutic INR’ alerts, which led to few interventions despite significant numbers of alerts thanks to the prescription security provided by corollary orders (INR are automatically ordered and displayed for each vitamin K antagonist prescription/dose adjustment) [43, 44].

### PharmaCheck’s impact on the activity of clinical pharmacists

The time required to process alerts was not accurately measured (estimated at 1 to 3 h per day, including reviewing new alerts and repetitive alerts occurring during several rounds of PharmaCheck use). Users nevertheless agreed on the need to allocate more time for analysis when alerts are first fired off (compared to an alert that has already been analyzed and requires a simple follow-up). Using PharmaCheck daily seemed to be a reasonable use of time—a means to avoid missing any important warnings resulting from new or changed prescriptions and potentially leading to an ADE. Indeed, it has been shown that when alerts cannot be analyzed daily, 36% of the notified situations handled retroactively (after 24 h) were associated with an ADE [29]. This suggests that screening would be most effective as part of daily routines.

Clinical PPV with alerts filtered by pharmacists (71%) were close to those observed when using similar screening tools (63% to 83% for 300 to 554 interventions) [30–32]. PharmaCheck was based on a back-office approach in addition to pharmacist’s participation during medical rounds. A majority of interventions were carried out by telephone as only a minority of situations involved patients admitted to a ward covered by a clinical pharmacist. A previous study showed a slightly higher final clinical PPV, around 80% [19]. In contrast, several studies have shown that acceptance rates for pharmacists’ interventions were significantly lower for back-office or written interventions than for on-ward interventions [19, 45]. Presence on a ward is more conducive to interventions as visibility and recognition are better. There is also better contextualization as the information used is captured during pharmacists’ visits [46]. Combining an on-ward approach with back-office screening and interventions for high-risk situations seems to us an effective and safe way to expand a clinical pharmacist’s coverage (and workload). The main reason (85.2%) physicians did not follow therapeutic adjustment proposals was a positive benefit–risk ratio. Although the risks associated with situations were explained during telephone calls with physicians, it was not always easy to weigh up the benefits from a distance, and without an initial discussion with the patient’s care team.

We used clinical PPV without a pharmacist’s intervention as a proxy measure of the impact PharmaCheck would have had if alerts were addressed to physicians. Under these circumstances, 14% of alerts would have been associated with a change in prescription—five times less than after pharmacists have filtered futile alerts. It is noteworthy that our approach assumes that interventions that led to a prescription modification would also have been recognized and led to the same modifications without a pharmacist. This optimistic assumption is consistent with results showing acceptance rates (i.e., prescription modifications) varying between 14.0 and 90.0% for alerts appearing as pop-ups on physicians’ digital interfaces during order entry [47]. Moreover, it should be noted that the clinical rules actively triggered when a caregiver consults the patient’s record at the time of prescription do not consider that the situation might evolve during hospitalization and require subsequent adjustment (before the patient’s file is consulted again). CDSS like PharmaCheck enable continuous passive monitoring. Thus, the prescription of a direct oral anticoagulant initiated several days before, associated with a sudden and brutal deterioration of renal function, will trigger an alert as soon as the laboratory results are published.

### Strengths and limitations

One of this work’s strengths is that our CDSS was developed locally and tailored to our needs. This allowed us to design queries adapted to our practices quickly (this was notably the case during the first wave of COVID 19 with the deployement of new queries in a few days) [48]. Thus, we were not confronted with the potential problems of interoperability with third-party software, which could have slowed down the CDSS’ deployment [35]. Moreover, specific functionalities could be added according to our needs (e.g., regarding renal function assessment, the ergonomics of alerts displays) and to explore certain aspects (e.g., search for keywords in patient files to characterize pathologies). Financially, having CDSS programing skills within the pharmacy was another strength. Indeed, despite lower development costs, our CDSS seemed to be equivalent to commercial rule-based systems, especially in terms of intervention PPV [25, 49]. This freed us from the financial constraints associated with acquiring and integrating a commercial CDSS [35]. The originality of this work lies in the centralization in the pharmacy of the ability to create a CDSS and to test and validate its functioning. It is worth noting that this was made possible despite the fact that no member of our team has any training in computer programming. We believe that the creation of CDSS by and for pharmacists allows us to meet specific needs and to gain in relevance. Finally, we elaborated a model to determine which types of electronic queries were the most effective and likely to result in a change in prescription. This understanding is important for identifying types of alerts—those likely to be sent to physicians at the prescribing stage (with a high clinical PPV without a pharmacist) and those likely to be directed more specifically to a pharmacist (with a low clinical PPV without a pharmacist but associated with a high clinical PPV with one).

This work also had some limitations. A large number of alerts were deemed irrelevant and no interventions were made. One of the main reasons for this over-representation is linked to the quality of the data relating to patients’ problems (the second most common alerts) and the insufficient specificity of some clinical rules. Recent reconfigurations of the clinical rules in our EHR, taking into account these structural problems, will certainly solve some of them [37]. Moreover, PharmaCheck’s specificity could be improved by adjusting queries to consider several discriminating conditions and by adding new triggers [50]. Another limitation is that the potential negative predictive values of the different clinical rules have not been assessed, as this would have created a heavy workload and required a manual chart review. However, except for patient problems, the triggers for PharmaCheck’s alerts are structured data (ATC codes, dosage values, biological analysis identifiers, etc.) that have been previously listed for query creation. Thus, these data are in our system permanently and we expect a low proportion of false-negative alerts. PharmaCheck can identify high-risk situations at a distance from the prescriber, but it may take several hours each days for the pharmacist to check for alerts (PharmaCheck runs at a fixed time on weekdays), meaning the occurrence of adverse events remains possible. Indeed, PharmaCheck complements a system currently being deployed that is based on alerts sent to physicians as they are prescribing drugs [51, 52]. An overall strategy will thus make it possible to consolidate prescription safety by combining multiple contextualized alerts, monitoring opportunities, and targeted healthcare professionals. Finally, the maintenance of our tool (adjustment of electronic queries, updating of the clinical knowledge base) will be a future challenge. This will require new pharmaceutical resources to be dedicated to this maintenance.

## Conclusions

With a relatively small investment in human resources, using PharmaCheck enables clinical pharmacists to expand their reach and screen every patient admitted to our General Internal Medicine Department for several high-risk situations. This tool, therefore, enhances our clinical pharmacy processes and boosts the efficiency of our clinical pharmacists. Our study underlined the importance of humanizing the management of alerts generated by a CDSS dedicated to pharmacists. This approach enabled the selection of the most relevant alerts to send to physicians and led to changes in prescriptions in most cases. Without filtering by a pharmacist, physicians would have had to identify the minority of relevant situations in a large number of alerts, a circumstance conducive to alert-fatigue and poor adherence to the system. A perspective to this work is the re-evaluation of the targeted risk situations. Outside the experimental framework, the definition of the situations sought should be the subject of a multi-disciplinary evaluation allowing the most relevant medication-related risks for our institution to be targeted. Also, PharmaCheck’s adoption by other departments (e.g., geriatrics, oncology, pediatrics) will involve the system being adapted to other specific populations for whom situations at a high-risk of adverse drug events must be characterized.

## Data Availability

The datasets analyzed during the current study are not publicly available due to institutional privacy and data sharing policies but a de-identified data set without any personal health information is available from the corresponding author on reasonable request if approval is granted by the institutional privacy office through a data use agreement and through the Institutional Review Board.

## Abbreviations

ADEs: Adverse drug events
AF: Atrial fibrillation
ATC: Anatomical therapeutic chemical
CDSS: A clinical decision support system
CG: Cockcroft–Gault
CKD-EPI: Chronic Kidney Disease Epidemiology Collaboration
ClCG: Estimated creatinine clearance using the Cockcroft–Gault formula
CPOE: Computerized physician order entry
CYP3A4: Cytochrome P450 3A4
DDI: Drug-drug interaction
DOAC: Direct oral anticoagulant
eGFR: Estimated glomerular filtration rate
EHR: Electronic health records
INR: International normalized ratio
IQR: Interquartile range
LMWH: Low molecular weight heparin
ME: Medication errors
MTX: Methotrexate
NSAID: Non-steroidal anti-inflammatory drug
P-gp: Permeability glycoprotein
PPV: Positive predictive value
PRN: As needed
SD: Standard deviation
UFH: Unfractionated heparin
VKA: Vitamin K antagonist
VTE: Venous thromboembolism
